# Status quo and future developments in the diagnosis and treatment of hereditary angioedema

**DOI:** 10.1111/ddg.15889

**Published:** 2025-09-04

**Authors:** Andreas Recke

**Affiliations:** ^1^ Department of Dermatology Allergology and Venereology University Hospital Schleswig‐Holstein – Lübeck Campus Lübeck Germany

**Keywords:** Bradykinin, C1 esterase inhibitor, hereditary angioedema, prophylaxis, therapy

## Abstract

Hereditary angioedema (HAE) is a rare hereditary disease characterized by edema, which can be life‐threatening in case of swelling in the larynx. The most common form of HAE is caused by a mutation of the *SERPING1* gene and is characterized by a deficiency (type I) or loss of function (type II) of the C1 inhibitor (C1‐INH), leading to excessive production of bradykinin. In contrast, the HAE‐nC1‐INH entity is associated with a normal C1‐INH protein and is caused by mutations in other genes. Because HAE is a rare and often underdiagnosed disease, it may take years from symptom onset to diagnosis. The angioedema attacks cause suffering and affect both the ability to work and quality of life (QoL). The treatment of HAE includes attack treatment (on‐demand), short‐term prophylaxis (e.g., before medical procedures), and long‐term prophylaxis. Four first‐line treatment options for long‐term prophylaxis are available, effectively preventing attacks and supporting the guideline goal of complete disease control. Further treatment options, including CRISPR/Cas9‐based gene therapy, are under development and promise to provide individually tailored treatment for patients. This review aims to provide an overview of the clinical presentation, diagnosis, and treatment of HAE.

## INTRODUCTION

Angioedema refers to a temporary swelling of the dermis, subcutis, or submucosa. It is caused by dilatation of blood vessels and increased vascular permeability due to vasoactive mediators such as histamine and bradykinin. Angioedema can occur spontaneously or result from a rare genetic disorder known as hereditary angioedema (HAE). The exact prevalence of HAE is unknown but is estimated to range from 1:50,000 to 1:100,000 people, depending on the region.^1^, ^2^ Patients with HAE experience recurrent episodes of non‐retractable edema, which can involve the tissues of the face, extremities, gastrointestinal tract, urogenital tract, and oropharynx. In rare cases, the larynx and hypopharynx may also be affected, which is life‐threatening for patients without treatment.^3^ A systematic review found that the estimated risk of death caused by asphyxia in patients with HAE was 8.6%.^4^ Bork et al. reported that untreated or undiagnosed HAE patients have a shortened lifespan of approximately 31 years.^5^ In addition to the direct impact on health, other aspects of life are also affected. For instance, many patients face limitations in their education or career choices due to HAE, which can result in financial loss.^1^ A study conducted in the European Union (EU) found that attacks, which can occur more than once a week in some patients, result in an average of 20 days of absence from work or school per year.^6^ For more than half of HAE patients, this average rises to 47 days.^7^ In addition to time lost from work and education – an average of 2.3 hours per week, according to a survey of members of the *US Hereditary Angioedema Association* (HAEA) – patients lose a further 3.2 hours per week for everyday activities, resulting in a corresponding decline in quality of life.^8^

## ETIOLOGY AND PATHOGENESIS OF HAE

The development of angioedema is primarily linked to two signaling pathways: the histaminergic signaling pathway, where mediators from basophilic granulocytes and mast cells play a crucial role, and the bradykinin‐dependent signaling pathway.^9^ For some variants of HAE, the vascular endothelial growth factor (VEGF) signaling pathway may also play a role.^10^ These findings gave rise to a new categorization of HAE variants in the *DANCE classification* (Definition, Acronyms, Nomenclature, and Classification of Angioedema). The categorization distinguishes between the bradykinin‐mediated (AE‐BK) and the vascular‐endothelial dysfunction‐mediated (AE‐VE) variants.^11^

**FIGURE 1:**
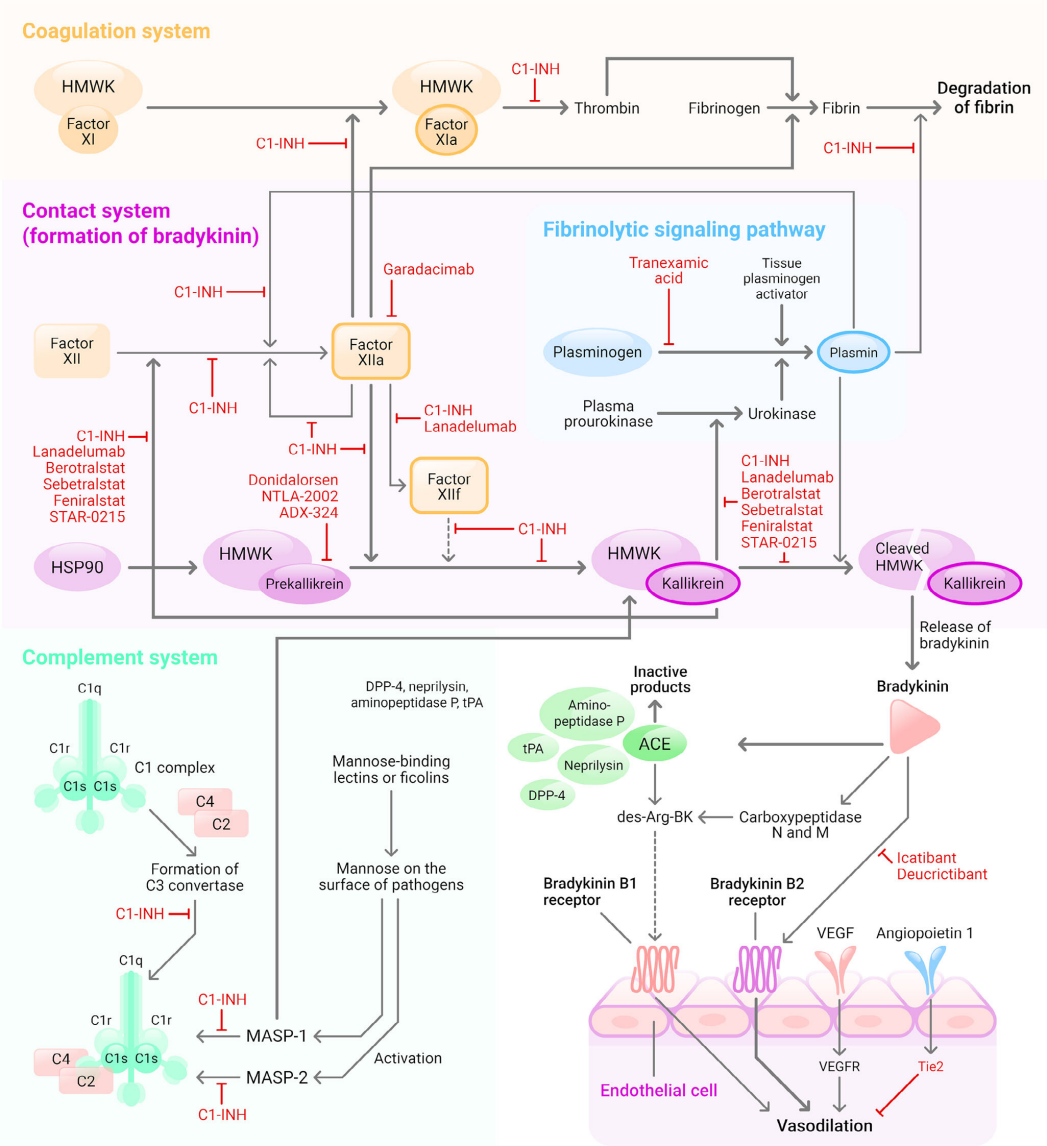
Possible role of the contact, complement, and coagulation system in the pathogenesis of HAE and possible therapeutic approaches. The figure was created using data from the KEGG PATHWAY Database (https://www.kegg.jp/kegg/pathway.html) and other information.^13^, ^51^, ^62^ Target sites of inhibition by C1‐INH or therapeutic substances are shown in red. The figure also shows how variants in FXII, PLG, HMWK (synonymously named KNG1), and ANGPT1, which are present in HAE‐nC1‐INH, are classified in the signaling cascade. Further variants that are present in HAE‐nC1‐INH are found in heparan sulfate‐glucosamine 3‐sulfotransferase 6 (HS3ST6) and myoferlin (MYOF), which are not shown here. The illustration was created using the Adobe InDesign program. *Abbr*.: ACE, angiotensin converting enzyme; ANGPT1, angiopoietin 1; Arg, arginine; BK, bradykinin; C, complement component; C1‐INH, C1 inhibitor; des‐ARG‐BK, des‐arginine‐bradykinin; DPP‐4, dipeptidyl peptidase‐4; FXII, coagulation factor XII; HAE, hereditary angioedema; HAE‐nC1‐INH, HAE‐associated with normal C1‐INH concentration and function; HMWK, high molecular weight kininogen; HSP90, heat shock protein 90; KNG1, kininogen 1; MASP, mannose‐binding lectin‐associated serine protease; PLG, plasminogen; Tie2, tyrosine kinase with immunoglobulin‐like loops and epidermal growth factor homology domains 2; tPA, tissue‐type plasminogen activator; UNK, unknown; VEGF, vascular endothelial growth factor; VEGFR, VEGF receptor

Bradykinin‐dependent HAE with C1 inhibitor (C1‐INH) deficiency, which represents the most common form of HAE, is caused by an autosomal dominant mutation of the serine protease inhibitor gene 1 (*SERPING1*). This results in either a deficiency (type I) or a functional disorder (type II) of C1‐INH in the plasma. Among its various functions, C1‐INH regulates the spontaneous activation of complement factor C1 (Figure 1, Table 1).

**TABLE 1:** Classification and description of the different forms of HAE.^14^, ^20^, ^21^, ^89^

Group	Classification	Description	Affected gene (inheritance)	OMIM‐number	Comment
HAE‐C1‐INH	HAE‐C1‐INH Typ I	C1‐INH deficiency	*SERPING1* (ad)	#106100	Reduced C1‐INH concentration and function^a^
	HAE‐C1‐INH Typ II	C1‐INH dysfunction	*SERPING1* (ad)	#106100	C1‐INH concentration normal up to increased and C1‐INH function^a^ reduced
	Not classified	Intermittent C1‐INH deficiency	*SERPING1* (ar)	#106100	C1‐INH reduced in concentration and function during attacks^a^
HAE‐nC1‐INH^b^	HAE‐F12	Factor‐XII GoF mutation	*F12* (ad)	#610618	Factor XII levels and activity are unchanged in the usual routine measurement procedures
	HAE‐PLG	Plasminogen GoF mutation	*PLG* (ad)	**#**619360	Plasminogen can cleave bradykinin independently of kallikrein;^49^ the plasminogen activity is unchanged in the standard routine measurement methods
	HAE‐ANGPT1	Angiopoietin 1 variant	*ANGPT1* (ad)	**#**619361	Mechanism unknown; presumably affects VEGF and bradykinin signaling pathways; alterations in the nailfold capillaries
	HAE‐KNG1	Kininogen 1 variant	*KNG1* (ad)	#619363	Mechanism presumably in the sense of a GoF mutation with increased cleavability of kininogen 1 (syn. HMWK^c^)
	HAE‐MYOF	Myoferlin variant	*MYOF* (ad)	#619366	Mechanism unknown; presumably affects VEGF and bradykinin signaling pathways; alterations in the nailfold capillaries
	HAE‐HS3ST6	Heparan sulfate‐glucosamine 3‐sulfotransferase 6 variant	*HS3ST6* (ad)	#619367	Mechanism unknown; presumably deficient intracellular storage of kininogen 1 and increased presentation on cell surfaces with increased cleavage
	HAE‐CPN1	Carboxypeptidase N	*CPN1*	n.n.	Proposed mechanism: accumulation of bradykinin and anaphylatoxin; important: patients had not only angioedema but also urticarial symptoms
	HAE‐DAB2IP	Disabled homolog 2‐interacting protein	*DAB2IP (ad)*	n.n.	Loss‐of‐function pathogenic variants lead to the impairment of the endothelial VEGF/VEGFR2 ligand system
	HAE‐UNK	Not known	Not known	n. n.	Umbrella term for HAE variants with unknown or yet unidentified genetic mutation

^a^ C1‐INH function reduced by < 50% of activity,^88^

^b^ C1‐INH concentration and function (activity > 50%) normal

^c^ KNG1 and HMWK are synonymous terms for the same molecule. Bradykinin is enzymatically cleaved from KNG1 by kallikrein and PLG

*Abbr*.: ad, autosomal dominant; ANGPT1, angiopoietin 1; ar, autosomal recessive; C, complement component; C1‐INH, C1 inhibitor; F12, coagulation factor XII; GoF, gain of function; HAE, hereditary angioedema; HAE‐nC1‐INH, HAE‐associated with a normal C1‐INH concentration and function; HMWK, high molecular weight kininogen; HS3ST6, heparan sulfate‐glucosamine 3‐sulfotransferase 6; KNG1, kininogen 1; MYOF, myoferlin; n.n., not yet assigned; PLG, plasminogen; SERPING, serine protease inhibitor gene; UNK, unknown; VEGF, vascular endothelial growth factor; OMIM, Online Mendelian Inheritance in Man (URL: https://omim.org)

For type I, more than 800 different mutations at various positions in the gene are currently known. The C1‐INH expressed by the healthy allele is affected by the defective C1‐INH protein due to a dominant‐negative effect, ultimately leading to reduced secretion of C1‐INH.^12^

In type II, a missense mutation in exon 8 is usually responsible for a change in the mobile loop, which impairs the ability of C1‐INH for protease inhibition.^12^, ^13^ The exact mechanism that leads to a dominant‐negative effect in HAE type II is so far unknown. Compared to HAE type I, the C1‐INH concentration in type II is often even increased. Ponard et al. furthermore describe a so‐called intermediate type, which is characterized by a reduced concentration of dysfunctional C1‐INH.^12^ This variant cannot be distinguished from HAE type I by laboratory analysis. Interestingly, HAE variants with intermittent deficiency of C1‐INH have also been identified. They follow an autosomal recessive inheritance pattern,^14^ and are associated with a variably reduced or normal C1‐INH concentration.

The above‐mentioned variants of HAE are associated with kinin and/or contact system dysfunction, excessive bradykinin production, localized vasodilation and increased vascular permeability together with associated swelling.^1^ C1‐INH is thus involved in the control of three interconnected signaling cascades. These are the kallikrein‐kinin system (contact system), the complement system (classical and lectin pathway), as well as the fibrinolytic system. An overview of the C1‐INH interactions is shown in Figure 1.^9^, ^13^, ^15^, ^16^

Moreover, HAE can be caused by other genetic variants associated with a normal C1‐INH protein (HAE‐nC1‐INH, formerly summarized under HAE type III). Specific causal genetic variants in HAE‐nC1‐INH have been identified in *F12* (coagulation factor XII), *ANGPT1* (angiopoietin‐1), *PLG* (plasminogen), *KNG1* (kininogen), *MYOF* (myoferlin), and *HS3ST6* (heparan sulfate glucosamine 3‐O‐sulfotransferase 6) (Table 1).^17^, ^18^, ^19^ Other genetic variants have recently been found in the *CPN1* gene (carboxypeptidase N) and in *DAB2IP* (disabled homolog 2‐interacting protein).^20^, ^21^ Nevertheless, in many cases of HAE‐nC1‐INH, the underlying genetic defect remains unknown.^13^, ^18^ The so‐called vibratory angioedema, that in some cases is caused by a mutation in the mucin‐like hormone receptor‐like receptor 2 (EMR2), which contains an epidermal growth factor‐like module and is encoded by the *ADGRE2* gene, is a special form of hereditary angioedema that is not (yet) classified as HAE.^22^, ^23^

**FIGURE 2:**
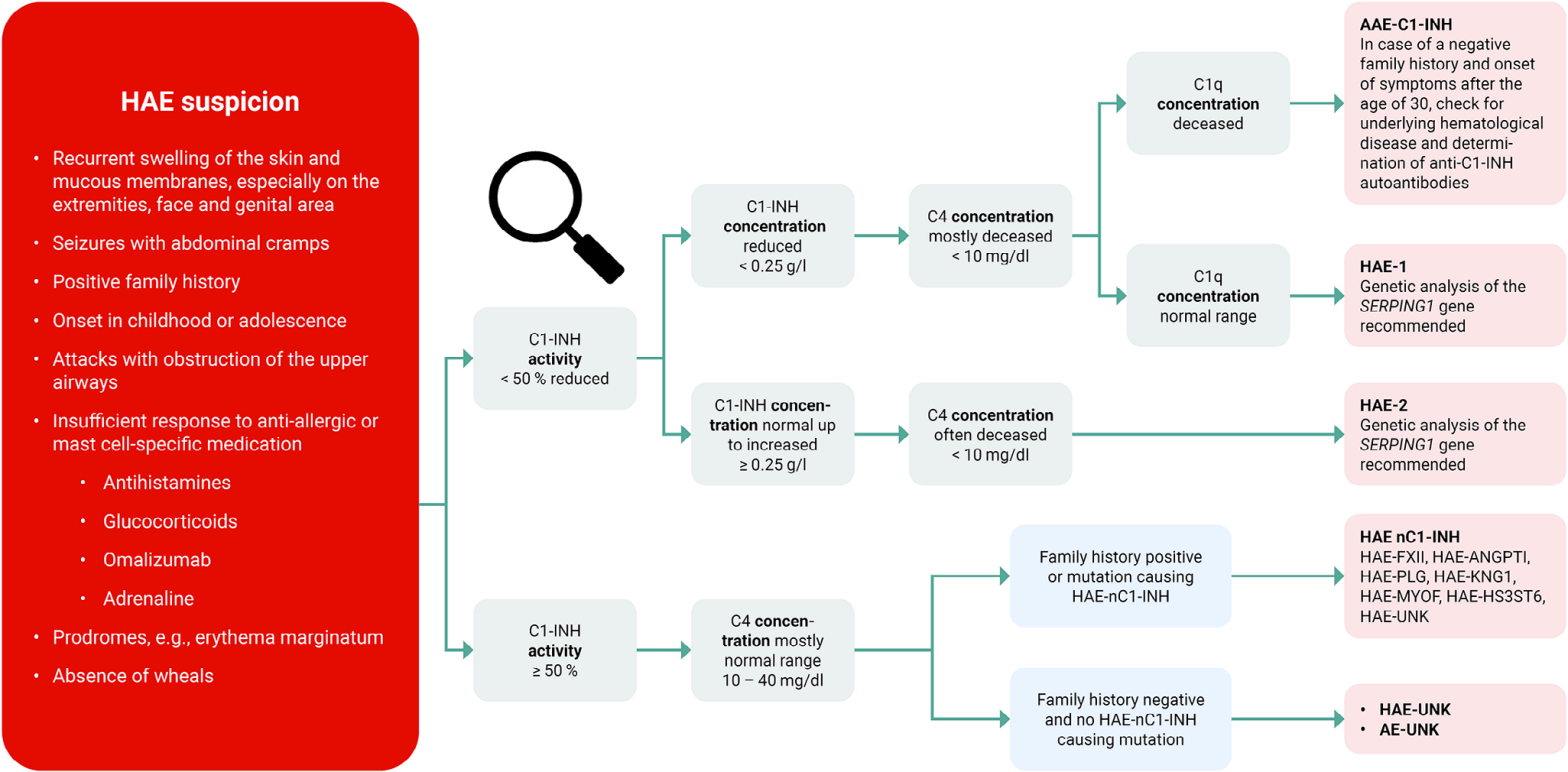
Diagnostic algorithm for patients with suspected HAE.28 The threshold of 50% activity of C1‐INH for diagnosis is derived from the study by Honda et al.88 There are no separate diagnostic threshold values for the concentration of the complement factors C1‐INH, C4, and C1q for the diagnosis of HAE or the differentiation between HAE‐1 and HAE‐2. The illustration was created using Microsoft PowerPoint. *Abbr*.: AAE‐C1‐INH, acquired angioedema due to C1‐INH deficiency; AE, angioedema; ANGPT1, angiopoietin 1; C, complement component; C1‐INH, C1 inhibitor; FXII, coagulation factor XII; HAE, hereditary angioedema; HAE‐nC1‐INH, HAE‐associated with normal C1‐INH concentration and function; HS3ST6, heparan sulfate‐glucosamine 3‐sulfotransferase 6; KNG1, kininogen 1; MYOF, myoferlin; PLG, plasminogen; UNK, unknown

The kallikrein‐kinin system is primarily responsible for the development of HAE symptoms. Activated factor XIIa triggers the cleavage and activation of the serine protease kallikrein, which then cleaves high molecular weight kininogen (HMWK), releasing bradykinin (Figure 2). The generation of bradykinin and its binding to the bradykinin β2 receptor results in the release of nitric oxide and other vasodilating factors from the endothelium. Lack of regulatory C1‐INH allows this process to be repeated and amplified without hindrance. Some of the other gene mutations in HAE‐nC1‐INH act in a similar way, ultimately releasing an excess of bradykinin. Consequently, the permeability of the vascular endothelium increases and fluid leaks into the extracellular space, leading to the development of angioedema.^9^ The swelling usually develops over several hours and disappears after 2 to 5 days if left untreated.^3^ However, the pathomechanism has not been fully clarified for all variants of HAE‐nC1‐INH.

Triggers for the activation of this signaling cascade and thus for the development of angioedema include trauma, surgery or dental treatment, stress, menstruation, use of oral contraceptives, or infections.^3^ For example, in a retrospective study with data from 247 HAE‐C1‐INH patients, perioperative angioedema occurred in 19% of participants who had not taken any prophylactic measures prior to surgery. As not all procedures documented the presence or absence of angioedema, the overall probability of occurrence is estimated at 5.7% to 30.5%. Swelling usually affected the area of the operation, although other localizations are possible. The development of angioedema was independent of the type of surgery.^24^ Similar findings were observed for tooth extractions. According to a retrospective study, angioedema developed in 21.5% of HAE patients following a total of 577 sessions in which one or more teeth were removed without HAE prophylaxis. In most of these cases, the swelling affected the face and, to a lesser extent, the larynx.^25^

## SYMPTOMS OF HAE

The angioedema can appear with a variety of prodromal symptoms (Table 2), which differ depending on the affected organ system. Initially, patients often report general symptoms such as fatigue, malaise, and influenza‐like symptoms without specific clinical diagnostic findings.

**TABLE 2:** Common symptoms by organ system.^90^

Organ system	Symptoms	Medical findings
General physical condition	Fatigue, malaise, influenza‐like symptoms	None
Upper respiratory tract	Globe sensation, dysphonia, dyspnea or dysphagia	Laryngeal edema, uvular edema or pharyngeal edema, tongue edema, hypoxemia
Cardiovascular system	Dizziness, heart palpitations	Hypotension, tachycardia
Skin and mucous membranes	Facial swelling, swelling of the lips or localized asymmetrical, non‐dependent swelling of the extremities without pruritus	Non‐local asymmetric edema, erythema marginatum (variable prodrome)
Gastrointestinal tract	Abdominal pain, nausea, vomiting, cramp‐like sensations, diarrhea	Intestinal edema, ileus, ascites
Urogenital tract	Genital pain, cramp‐like sensations	Swelling in the genital area
Neurological system	Hypesthesia, paresthesia at the sites of the impending attacks	Reduced sensorial sensitivity

Typically, there is swelling of the face and lips, but also local swelling of the extremities or genital area is possible. A particular skin symptom is the erythema marginatum, which occurs in some patients as a sign or prodromal symptom of an attack.

The swelling of the tongue, uvula, pharynx, or larynx can lead to dyspnea, dysphagia, and dysphonia. In severe cases, there is a risk of complete airway obstruction with subsequent hypoxemia. This highlights the importance early intervention – including cricothyrotomy in critical cases – to ensure airway patency.

When the gastrointestinal tract is involved, patients suffer from abdominal pain, nausea, vomiting, and cramp‐like sensations. These symptoms are often accompanied by intestinal edema, ileus, or ascites, which can further complicate the course of the disease. They are frequently mistaken for an “acute abdomen”, leading to unnecessary laparotomies. As a result, many patients have abdominal scars from these procedures.

The genitourinary tract can also be affected, causing pain and cramp‐like sensations in the genital area. In such cases, localized swelling is clinically observable.

Hypoesthesia and paresthesia as well as fatigue, malaise, mood swings, myalgia, and arthralgia may precede attacks.^26^

## DIAGNOSIS OF HAE

In addition to clinical symptoms – such as skin swelling, laryngeal oedema, ascites, and gastrointestinal issues like abdominal pain, nausea, and vomiting – the family history is an important factor in the diagnosis of HAE. HAE‐C1‐INH is usually inherited in an autosomal dominant manner, meaning that almost all affected individuals are heterozygous. Medical associations therefore recommend testing children with a positive family history as early as possible, especially those with affected parents. However, the disease has a high rate of spontaneous mutations (20%–25%), so a negative family history does not completely rule out HAE‐C1‐INH.^26^, ^27^, ^28^ Other key indicators include symptom onset during childhood or adolescence and a lack of response to antihistamines, glucocorticoids, omalizumab, or epinephrine.^28^

HAE‐C1‐INH is characterized by the absence of pruritus and urticaria and can therefore be ruled out if these symptoms are present. For the diagnosis of HAE‐C1‐INH, laboratory measurement of the C1‐INH protein with testing of the concentration and function in serum/plasma and measurement of the concentration of C4 complement in blood samples is recommended. C1‐INH function is typically reduced to below 50% of the normal value, accompanied by decreased C4 levels. In type I, C1‐INH concentration is also reduced to below 50% of the normal value, whereas in type II, it may remain unchanged or even elevated. Analyzing these three parameters together allows for a highly accurate diagnosis of the disease.

To further confirm the diagnosis, sequencing of *SERPING1* can be performed.^26^, ^28^ In cases requiring comprehensive genetic analyses, consulting experienced specialists at an HAE center is recommended. Their expertise supports both accurate diagnosis and informed treatment decisions.

In HAE‐nC1‐INH, C1‐INH and C4 levels are normal, making genetic analysis essential for diagnosis. According to the current state of the art, whole‐exome or even whole genome sequencing is recommended.^29^ Depending on the method used, it is possible to detect structural gene changes such as copy number variations, as well as exon deletions and exon multiplications, in addition to short insertions/deletions and base substitutions. For certain genes, multiplex ligation‐dependent probe amplification (MLPA) is also available, offering particularly high precision and sensitivity in identifying those structural gene changes.^30^ However, interpreting the results requires substantial expertise in human genetics.

For detection of coagulation factor XII (FXII) HAE, only exon 9 of the factor XII gene (*F12*) should be examined in routine diagnostics, as four known mutations can be found there. However, only 20%–25% of HAE‐nC1‐INH patients in Europe show an *F12* mutation, which is why other genes often also need to be tested (Table 1). These include the genes for angiopoietin‐1 (*ANGPT1*), plasminogen (*PLG*), kininogen‐1 (*KNG1*), myoferlin (*MYOF*), and *HS3ST6*.^19^, ^28^, ^31^ An algorithm for the diagnosis of angioedema is shown in Figure 2.

It is possible that none of the aforementioned methods will detect a gene variant confirming the HAE‐nC1‐INH diagnosis. In such cases, a systematic approach and focus on warning signals (red flags) for bradykinin‐mediated angioedema can be helpful. These are described in the guideline,^28^ of the *World Allergy Organization/European Academy of Allergy and Clinical Immunology* (WAO/EAACI): (1) a positive family history, although this is absent in up to 25% of patients; (2) onset of symptoms in childhood or adolescence; (3) recurrent and painful abdominal symptoms; (4) edema with upper airway obstruction; (5) lack of response to antihistamines, glucocorticoids, omalizumab, or epinephrine; (6) prodromal symptoms or signs, such as transient erythema (erythema marginatum), hypoesthesia, paresthesia, and emotional changes prior to swelling; (7) absence of wheals.

When these warning signs are present, the diagnosis of hereditary or bradykinin‐mediated angioedema should not be dismissed lightly, even if the genetic testing is negative. Buttgereit and Magerl describe a systematic approach for diagnosing HAE Unknown (HAE‐UNK) in such cases.^32^ In particular, lack of response to standard antihistamine therapy at four times the usual dose, as well as to omalizumab, in combination with a corresponding family history, is regarded as a strong indicator of HAE. However, the family history may be negative in the case of somatic or new mutations, which are seen in 25% of HAE‐C1‐INH cases.^28^ In such patients, the diagnosis of idiopathic angioedema is formally made, raising the unresolved question of whether a variant of angioedema exists that could respond to the treatment options for hereditary angioedema.

## IMPACT ON QUALITY OF LIFE

HAE has a significant impact on most areas of the patient's life. Several disease‐specific questionnaires such as the *Angioedema Activity Score* (AAS),^33^ the *Angioedema Quality of Life Questionnaire* (AE‐QoL),^34^ the *Angioedema Control Test* (AECT),^35^ and the HAE‐QoL,^36^ have been developed to assess disease activity and quality of life (QoL).^37^

The *AAS* was developed in Germany in 2013 and is one of the most relevant scores for assessing HAE disease activity. It is recommended by the guidelines as the standard for assessing and monitoring patients.^28^ A score is obtained by answering five questions, which indicates either low or high disease activity. The assessment can take place over 7, 28, or 84 consecutive days.^33^, ^38^

The first questionnaire designed for patients with HAE was the *AE‐QoL*, which correlates well with angioedema attacks. However, if attacks occur very frequently, it reaches a plateau value, making it less suitable for comparing patients with varying levels of disease activity. Furthermore, the correlation is influenced by psychological factors such as anxiety about recurrent attacks, so that reduced disease activity may be associated with a lower level of improved QoL than expected.^34^

The *AECT* is used to test the disease control experienced by the affected patients. The test takes into account the number of angioedema attacks, their impact on QoL, the degree of concern caused by the unpredictability of the attacks, and how well the angioedema is controlled by the therapy. The aspects are assessed on a scale ranging from 0 to 16 (higher = better), with a score of at least 10 indicating that the disease is well controlled.^35^

The *HAE‐QoL* questionnaire was developed for adults with HAE‐C1‐INH and is one of the most specific tools for assessing QoL in this cohort. It covers 25 items from the following seven domains: treatment difficulties, psychological functioning and health, disease‐related stigma, emotional role and social functioning, worries about passing on to offspring, experienced disease control, and mental health. In total, between 25 and 135 points can be achieved, with a higher score indicating a better QoL.^36^

A consensus report on the assessment of QoL for patients with HAE is available for Europe and the USA. This report outlines recommended approaches, including specific questions to evaluate the disease burden (Table 3). The guidelines emphasize that QoL assessments should be individualized, considering the unique impact of HAE on the patient's work, school, social and family life, physical activity, treatment access, and the burden of treatment.^39^

**TABLE 3:** Recommended questions to evaluate the burden of disease in HAE patients.^39^

Are there any activities that you avoid because of your HAE?
How often do you experience HAE attacks?
How would you describe the severity of your HAE attacks? (0 = no impairment; 4 = total disability)
How often are you absent from work, school or unable to carry out activities at home due to HAE?
How often do you have to take acute emergency medication for each HAE attack? Do you feel that you are responding well?
What is the average time from the onset of the attack to the administration of treatment? Time to first symptom relief? Time to complete disappearance of symptoms?
Have there been any changes in your life that could affect the activity of your HAE?
How often do you experience fear, anxiety, or depression associated with your HAE?
Do you have difficulty maintaining or completing your acute or prophylactic HAE treatment?
To what extent has HAE affected your social life, your family, your relationships or your physical activities?
How many times have you been hospitalized due to an HAE attack?
Have you changed your lifestyle to avoid triggers of attacks?

*Abbr*.: HAE, hereditary angioedema

A web‐based survey on QoL and disease‐related impact on life conducted in 2017 among 445 members of the US *Hereditary Angioedema Association* (HAEA) using several QoL instruments showed that HAE‐C1‐INH is still a significant burden on the daily lives of patients despite the available treatment options.^8^ Participants reported an average of 11.1 attacks within 6 months.^8^ 78.7% had an attack in the last month and 41.8% in the last week, with the majority of participants classifying the symptoms of their last attack as moderate or severe.^8^ Health‐related QoL (HRQoL), anxiety (49.9% of participants), and depression (24%) increased with the frequency of attacks and had a significant impact on overall QoL scores.^8^ Patients without anxiety had a high score of 106 points on average in the HAE‐QoL questionnaire,^36^ while those with severe anxiety had a score of 62.1 points.^8^ Without depression, the average HAE‐QoL score was 100 points, while patients with severe depression had scores of around 51 points.^8^ Increased anxiety symptoms were already detectable in children and had the same negative impact on QoL as in adults.^40^, ^41^

HAE symptoms impair productivity at work and other everyday activities.^6^ In addition, health and emotional problems such as pain, fatigue, or depression make it difficult for patients to participate in social activities.^8^, ^42^ Many patients feel forced to avoid traveling and certain hobbies, and sports are also only possible to a limited extent.^43^ Another relevant factor that contributes to reduced QoL is the fear of passing HAE on to one's own children. For this reason, many of the affected people decide not to have children or to have fewer children than desired.^44^

**FIGURE 3:**
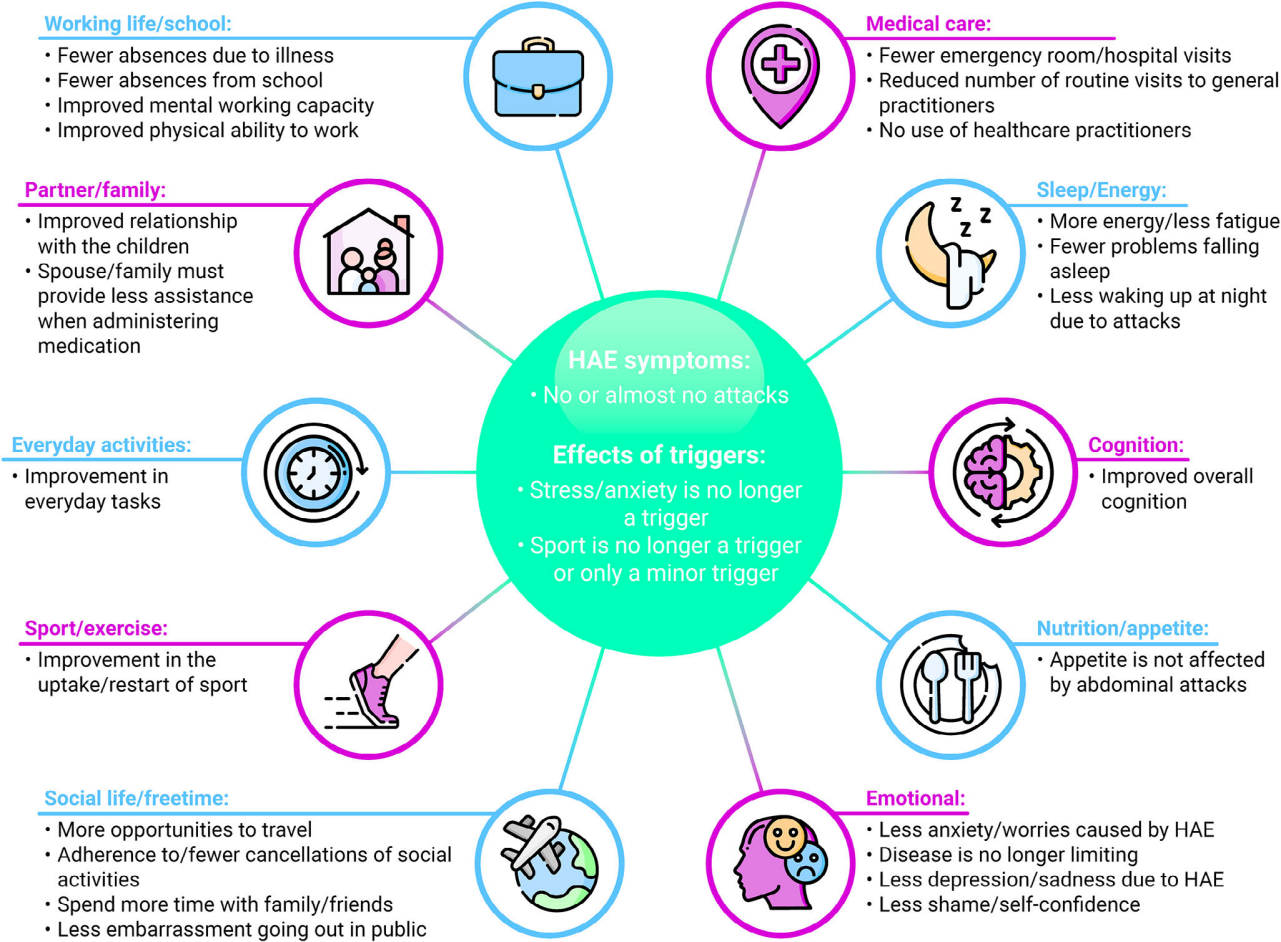
Concept model of improving HRQoL by preventing attacks using long‐term prophylaxis.^37^ The illustration was created using the program Adobe InDesign. *Abbr*.: HAE: hereditary angioedema; HRQoL: health‐related quality of life

Personalized therapy can address the above‐mentioned aspects and partially resolve problems (Figure 3). The use of a pharmaceutical prophylaxis can improve QoL compared to on‐demand therapies alone.^45^

Associations such as the *HAE‐Vereinigung e.V*., the international patient organization *HAE International* (HAEi), and the HAEA provide patients with information, opportunities to share experiences, and access to the latest information and treatment options, thereby making a significant contribution to improving patients' QoL.

## OVERVIEW OF GUIDELINES AND THERAPEUTIC APPROACHES

Various professional societies have published guidelines for the diagnosis and treatment of HAE, including the German S1 guideline (currently under revision)^27^ and the international WAO/EAACI guideline.^28^ Furthermore, the *DANCE classification* was developed as a supplement to the clinical guidelines through an initiative involving experts from various countries. The purpose of the revised classification and nomenclature of HAE is to enable a more standardized and systematic approach to clinical studies, which can contribute to improved diagnosis and treatment of patients.^11^

The goal of the HAE treatment recommendations is to achieve the best possible, or ideally, complete control of the disease. If an attack occurs, the use of on‐demand medication should therefore always be considered. In case of potential laryngeal involvement, treatment is absolutely indicated. The primary goal of the on‐demand therapy is to reduce swelling in the affected areas, which can be life‐saving due to the risk of suffocation, particularly if the angioedema is close to the throat. As the duration of the attack and the associated symptoms are shortened the earlier treatment is administered, it should be initiated as soon as possible.^28^ For this reason, patients should ensure that they always have sufficient medication on hand for self‐administration for at least two attacks.

Intravenous C1‐INH concentrates and the selective bradykinin B2 receptor antagonist icatibant are suitable first‐line treatment options for acute attacks of HAE‐C1‐INH. If these are not available, solvent detergent‐treated plasma (SDP) should be used. If this is also not possible, frozen fresh plasma serves as an alternative.^28^ However, anti‐fibrinolytic agents such as tranexamic acid, androgens such as danazol, corticosteroids, antihistamines, as well as epinephrine and its derivatives are unsuitable, as they provide little or no benefit in on‐demand treatment.^27^, ^28^

There are two different forms of C1‐INH concentrate, both of which act in the same way. This temporarily compensates for the C1‐INH deficiency, allowing C1‐INH‐dependent signaling cascades to proceed normally. The concentrates are either isolated from human plasma (pdC1‐INH) or produced recombinantly in transgenic rabbits (rhC1‐INH).^28^ In a double‐blind randomized study with 125 participants, the effect of pdC1‐INH at doses of 10 U/kg body weight (BW) and 20 U/kg BW was compared with placebo. Patients treated with 20 U/kg BW showed a significantly faster reduction in symptoms compared to placebo (on average 0.5 vs. 1.5 hours) and the time to complete resolution of symptoms was significantly shorter (4.9 vs. 7.8 hours). Moreover, C1‐INH concentrate was well tolerated. Treatment‐related adverse events occurred in 10.9% of participants after treatment with 20 U/kg and in 19.5% with placebo, with nausea being the most common event (6.5 vs. 12.2%).^46^ Similar results with regard to efficacy were obtained in a study with rhC1‐INH, where symptoms were reduced on average after 1.5 hours vs. 5.5 hours with placebo.^47^

Icatibant is injected subcutaneously and has been shown to be effective in two multicenter phase III studies. A total of 130 participants were randomized to receive either icatibant, tranexamic acid, or placebo for an acute cutaneous or abdominal attack. The primary endpoint was the mean time to significant improvement of index symptoms, which was reached after 2.5 and 4.6 hours for the comparison of icatibant with placebo, and after 2.0 and 12.0 hours for the comparison of icatibant with tranexamic acid, respectively. An initial improvement of symptoms was already observed by the participants after 0.8 hours with icatibant, while this was seen significantly later with placebo (16.9 hours) or tranexamic acid (7.9 hours).^48^

As the primary goal of HAE therapy is to control the disease and prevent attacks, patients should receive short‐term prophylaxis during planned medical procedures. Intravenous pdC1‐INH preparations are particularly suitable for this purpose and are therefore recommended by the *WAO/EAACI guideline* as first‐line treatment. In the second line, frozen fresh plasma can be administered. However, this treatment has a lower safety profile due to an increased risk of blood‐borne diseases or allosensitization. Attenuated androgens were considered as an alternative in the past – however, as they can induce side effects when used frequently, intravenous pdC1‐INH preparations are preferred.^28^ Whether short‐term prophylaxis is clinically necessary in addition to long‐term prophylaxis has not yet been finally clarified.^28^, ^49^ Sufficient access to the required medication and its rapid use as soon as the attack is recognized are therefore crucial factors.^50^

The treatment of HAE‐nC1‐INH is currently the same as for HAE‐C1‐INH. There is evidence that long‐term therapy with a kallikrein inhibitor is not effective in all patients; in HAE‐PLG, a kallikrein‐independent mechanism can lead to the production of bradykinin.^51^ For HAE‐MYOF and HAE‐ANGPT1, no therapeutic approaches have been described so far. Additionally, these variants appear to have a significantly different pathomechanism compared to other forms of HAE.^52^, ^53^

### Status of long‐term prophylaxis

Long‐term prophylaxis aims to generally prevent HAE attacks and the associated health risks while also preventing the loss of QoL and ability to work due to the disease burden. Effective long‐term prophylaxis gives affected people a greater sense of safety and enables them to live a more normal life.^54^

For long‐term prophylaxis, the *WAO/EAACI guideline* recommends pdC1‐INH, lanadelumab, and berotralstat as first‐line therapy. Given the half‐life of pdC1‐INH, it is typically administered twice weekly; for optimal efficacy, both frequency and dose should be individually adjusted.^28^ In contrast to on‐demand medication or short‐term prophylaxis, long‐term prophylaxis with C1‐INH can also be administered subcutaneously instead of intravenously. Subcutaneous application was investigated in a study with 79 participants. Both doses tested (40 and 60 international units [IU]) were effective, reducing the monthly HAE attack rate compared with placebo. The need for medication was significantly reduced from 5.55 applications per month in the placebo group to 1.13 applications in the 40 IU group and from 3.89 applications per month to 0.32 applications in the 60 IU group, respectively. In the C1‐INH groups, adverse events occurred to a similar degree as in placebo‐treated patients.^55^

The monoclonal antibody lanadelumab is also recommended as first‐line therapy for long‐term prophylaxis.^28^ It is usually administered every 14 days via subcutaneous injection of 300 mg and targets active plasma kallikrein. In an open‐label phase III extension study, 37.3% of the 209 participants treated with lanadelumab were attack‐free over a mean observation period of 29.6 months. Within the first 6 months, 18.2% of patients experienced attacks, and within the first 12 months, 31.1% of patients were affected. Treatment‐related adverse events occurred in 54.7% of participants, with pain at the injection site (42.5%) and redness of the skin (16%) being the most common adverse events.^56^ The efficacy and safety of lanadelumab are currently being evaluated in two long‐term studies (ENABLE; NCT04130191 and EMPOWER; NCT03845400). A retrospective longitudinal analysis based on anonymized healthcare data also showed that HAE patients may benefit from prophylactic treatment with lanadelumab in clinical practice. Of the 144 patients whose data were included in the study, the majority received only on‐demand medication prior to lanadelumab therapy. The results showed that approximately half of the patients receiving lanadelumab were able to switch to an extended treatment interval and required significantly less on‐demand medication. The results indicate that patients receiving lanadelumab can achieve a higher degree of disease control.^57^

Since approval of the plasma kallikrein inhibitor berotralstat in April 2021, an oral treatment option is available for long‐term prophylaxis.^28^ The effect of berotralstat was investigated in a three‐part phase III study (APeX‐2; NCT03485911). After 48 weeks of treatment with a daily dose of 150 mg berotralstat, a reduction in attacks from 3.06 per month at the start of the study to 1.06 was observed.^58^ The 96‐week data from 21 patients who were treated with 150 mg berotralstat (once daily) over the entire period provide a positive assessment of long‐term efficacy and safety. In addition to good tolerability, the participants showed a low mean monthly number of attacks of 0.33. Furthermore, QoL, treatment satisfaction, and the frequency of taking on‐demand medication improved.^59^ Safety and efficacy are still being investigated in a long‐term study (APeX‐S; NCT03472040). At the 48‐week interim analysis, the attack rate had decreased to 0.8 per month. Observed treatment‐related adverse events were mostly considered mild or moderate. The most common were abdominal pain (9.7%), diarrhea (7%), and nausea (6.2%).^60^

Attenuated androgens can be used for second‐line therapy, although they are no longer approved for this indication in Germany due to side effects, contraindications, and the potential for abuse and must therefore be obtained from an international pharmacy.^27^, ^28^ An overview of the approved medications for the treatment and prophylaxis of HAE attacks in the EU is shown in Table 4.

**TABLE 4:** Approval status of current HAE therapies.^91^, ^92^

Active substance	pdC1‐INH	rhC1‐INH	Icatibant	pdC1‐INH	Lanadelumab	Garadacimab	Berotralstat
*Application*	*IV*	*IV*	*SC*	*SC*	*SC*	*SC*	*Orally*
*Acute attack, age (years)*							
>18	+	+	+	–	–	–	–
12–17	+	+	+	–	–	–	–
6–11	+	+	+	–	–	–	–
2–5	+	+	+	–	–	–	–
*Short‐term prophylaxis*	+^*^	–	–	–	–	–	–
*Long‐term prophylaxis*							
>12	+^*^	–	–	+	+	+	+
6–11	+^*^	–	–	–	+	–	–
2–5	–	–	–	–	+	–	–

* Approval differs depending on the company. +, approved; −, not approved

*Abbr*.: HAE, hereditary angioedema; C, complement component; IV, intravenous; pdC1‐INH, plasma‐derived C1 inhibitor; rhC1‐INH, recombinant human C1 inhibitor; SC, subcutaneous

With the treatment options approved in recent years, long‐term prophylaxis is becoming increasingly important in HAE management, as it provides a basic level of protection against attacks. A study conducted in Leipzig, Germany, with 37 HAE patients compared QoL between on‐demand therapy and long‐term prophylaxis. Notably, in the categories of anxiety and depression – measured using the specific Generalized Anxiety Disorder‐7 (GAD‐7) and Hospital Anxiety and Depression Scale (HADS) questionnaires – long‐term prophylaxis led to a significant improvement in both areas. The disease control measured by AECT and the QoL determined by the AE‐QoL were also considerably improved with long‐term prophylaxis than with on‐demand therapy.^54^

The benefits of various treatments used for long‐term HAE prophylaxis were examined in a Cochrane Review. The analysis included 15 studies with 912 participants who received different prophylaxis therapies compared to placebo. The results suggest that C1‐INH, berotralstat, danazol (currently not available in Germany), and lanadelumab effectively reduce the risk and/or frequency of HAE attacks. Additionally, C1‐INH (all types), berotralstat, and lanadelumab improve patients' QoL compared to placebo while maintaining an acceptable safety profile.^61^

In patients with HAE and additional comorbidities that require medical treatment, it should always be checked in advance whether the medication used poses a health risk. Some drugs can affect the bradykinin signaling pathway and thus trigger angioedema attacks in HAE patients and should therefore be avoided. These drugs include estrogen‐containing preparations, ACE inhibitors, angiotensin II receptor blockers, renin inhibitors, neprilysin inhibitors, DPP‐4 inhibitors, and tissue plasminogen activators. Use of the aforementioned drugs can also lead to angioedema without the presence of HAE, a condition classified as drug‐induced angioedema (AE‐DI) according to the DANCE classification.^11^, ^62^

### Future perspectives in HAE therapy

Although effective therapeutic options for HAE are now available, there remains an unmet need for additional treatments. One approach is the use of the antisense oligonucleotide PKKRx, also known as donidalorsen, which inhibits plasma kallikrein expression at the messenger RNA (mRNA) level. Following promising results in the phase I and phase II studies,^63^, ^64^ results of the placebo‐controlled phase III study (NCT05139810) were recently published.^65^ Patients received donidalorsen or placebo at 4‐ or 8‐week intervals. Over 25 weeks, the donidalorsen groups achieved mean reductions in attack rate of 81% and 55%, respectively, compared with placebo. In addition, the median attack rate compared to baseline improved by 90% in the 4‐week donidalorsen group and by 83% in the 8‐week donidalorsen group, while the placebo group only achieved a reduction of 16%. In addition, the 4‐week treatment with donidalorsen had a positive impact on the patients' QoL. At the same time, the safety profile was acceptable.^65^, ^66^

ADX‐324 follows a similar strategy, using RNA interference technology (NCT05691361) to prevent HAE attacks. Initial interim results of the phase I study showed a significant reduction in the prekallikrein concentration in participants over a period of at least 3 months. The results are indicative for a safe treatment.^67^

Other therapeutic options are still in development. One example is the bradykinin B2 receptor antagonist deucrictibant, which is being evaluated in two phase II studies (NCT04618211, NCT05047185) for both the treatment of acute attacks and the prophylactic management of HAE.^68^, ^69^, ^70^ Initial results have shown that deucrictibant provides rapid and significant symptom relief, leading to a fast resolution of HAE attacks. Additionally, the substance was well tolerated at all tested doses.^71^ In a separate phase II study, deucrictibant also proved to be promising as a potential HAE prophylactic therapy, significantly reducing the frequency of HAE attacks compared to placebo and while maintaining good tolerability in both tested doses.^72^

In a phase III study (NCT04656418), garadacimab, a human monoclonal antibody that inhibits activated FXIIa, was tested for efficacy and safety as a prophylactic therapy in 65 HAE‐I/II patients. During the 6‐month period of treatment, garadacimab led to a significant reduction in the mean number of attacks per month compared to placebo, with a mean difference of –87%. The safety profile of garadacimab was favorable. In particular, the inhibition of FXIIa had no effect on the bleeding risk or the risk of thromboembolic events.^73^ As of February 2025, the drug has been approved in the European Economic Area and Japan and is now available for the treatment of HAE (Table 4).

In addition, the plasma kallikrein inhibitor ATN‐249, which is used orally for prophylaxis, is currently under development. In a phase I study with 48 participants, ATN‐249 was able to effectively reduce plasma kallikrein activity within 2 hours when administered daily *ex vivo*.^74^ The humanized immunoglobulin G1 (IgG1) antibody STAR‐0215 also inhibits the plasma kallikrein activity and is intended for use as HAE prophylaxis in the future. It is currently being tested in a phase I study (NCT05477160) with regard to efficacy, tolerability, immunogenicity as well as pharmacokinetic and pharmacodynamic parameters.^75^, ^76^ In initial results, the substance showed a favorable safety profile, a long half‐life and a sustained pharmacodynamic efficacy in healthy participants, making it a promising candidate for HAE therapy.^77^

For on‐demand therapy, the selective plasma kallikrein inhibitor sebetralstat (KVD‐900) was investigated in a phase III study (NCT05259917). Participants received up to two oral doses (300 or 600 mg) of sebetralstat or placebo when angioedema attacks occurred. Key endpoints included onset of symptom relief and reduction in severity of attacks, which were assessed in a time‐to‐event analysis. Patients taking sebetralstat experienced significantly faster symptom relief than patients taking placebo (1.61 and 0.78 to 7.04 and 1.79 vs. 6.72 hours). Additionally, the time to reduction of attack severity was shorter in patients taking sebetralstat than in patients taking placebo (median duration: 9.27 and 7.75 hours vs. > 12 hours).^78^, ^79^

A comparable compound is feniralstat (KVD824), which has been evaluated in a phase I study (NCT05178355). However, the subsequent phase II study, which evaluated the drug as a potential candidate for HAE prophylaxis, was terminated early for safety reasons, and further development of the compound was discontinued.^80^

Special developments include the *Clustered Regularly Interspaced Short Palindromic Repeats* (CRISPR)/Cas9‐based gene therapy NTLA‐2002, which targets the plasma kallikrein gene *KLKB1* and is intended to be used for HAE prophylaxis.^81^ NTLA‐2002 is currently being tested in a phase I/II study (NCT05120830) in adult HAE patients to evaluate its safety, tolerability, pharmacokinetics, and pharmacodynamics.^82^ Initial study results have already shown that NTLA‐2002 was both well tolerated and effective. Administration resulted in a dose‐dependent reduction in plasma kallikrein concentration and led to a reduction in the number of HAE attacks per month.^83^

Another gene therapy called BMN‐331 is based on an adeno‐associated virus 5 vector (AAV5), which leads to prolonged expression of C1‐INH and was investigated in a phase I/II study (NCT05121376). However, the study was terminated prematurely in 2024.^84^

## CONCLUSIONS

Hereditary angioedema is a rare hereditary disease that can become life‐threatening for patients if edema occurs in the larynx. In type I and type II HAE, the disease is caused by a deficiency or functional impairment of C1‐INH due to genetic defects in the *SERPING1* gene. This leads to attacks characterized by excessive release of bradykinin, which mediates edema formation. In contrast, HAE‐nC1‐INH is caused by mutations in other genes that do not result in specific changes in C1‐INH or other laboratory‐analyzable biomarkers. The diagnosis of HAE is often challenging due to its rarity, non‐specific symptoms, and the need for specialized diagnostic expertise – particularly in cases of HAE‐nC1‐INH. As a result, the disease is frequently overlooked in differential diagnoses. Studies show that the diagnostic delay can be substantial, with a median delay of 7 years in a report from Belgium,^85^ 15 years according to a report from Germany,^86^ and an average of 20.7 years in patients without a family history in a report from Portugal.^87^ The primary reasons for this delay include low awareness of the disease, its clinical similarity to other forms of angioedema, and the tendency of affected patients to postpone seeking medical attention Recurrent attacks often lead to loss of work capacity, adversely impacting education, career prospects, and daily life. This is associated with a significant reduction in QoL. Although the attacks can be controlled with on‐demand medication, prophylaxis is generally advisable to reduce their occurrence. Short‐term prophylaxis should be used for known triggers such as medical interventions. In addition, general long‐term prophylaxis can help to reduce the burden of disease and thus enable a normal life. Four efficient prophylactic therapy options are currently available for this purpose, with several new therapy options such as CRISPR/Cas9‐based gene therapy which are already very advanced in their development and could expand the range of treatment options in the future. As a result, individualized therapies according to the specific characteristics and underlying mutations will be possible in the future.

## FUNDING

Medical writing support and graphic design for this manuscript were provided by BioCryst Pharma Deutschland GmbH. The article was not reviewed or edited by BioCryst Pharma Deutschland GmbH.

## CONFLICT OF INTEREST STATEMENT

A.R. is an employee of the University Medical Center Schleswig‐Holstein, public law institution. He has received lecture and consulting fees, support for conference travel, and sponsorship for conferences organized on behalf of the University Medical Center Schleswig‐Holstein, Lübeck Campus, from Takeda Pharmaceuticals Company Ltd, BioCryst Pharma Deutschland GmbH, Pharming Group NV, and CSL Behring GmbH. A.R. declares that he did not receive any payment or other benefits from BioCryst for writing this manuscript. The idea for this review article was originally initiated by BioCryst. A.R. developed the idea further and independently prepared a comprehensive review article for the JDDG, including clarification of the conditions for writing a review article funded by the company. In the preparation of the manuscript, A.R. was supported by a medical writer and a graphic designer funded by BioCryst. A. R. undertook the following tasks: conceptualization, manuscript preparation, manuscript revision, supervision with a special focus on scientific neutrality, and the review.
